# Fabrication of (111)-Oriented Nanotwinned Au Films for Au-to-Au Direct Bonding

**DOI:** 10.3390/ma11112287

**Published:** 2018-11-15

**Authors:** John A. Wu, Chih-Yang Huang, Wen-Wei Wu, Chih Chen

**Affiliations:** Department of Materials Science and Engineering, National Chiao Tung University, Hsinchu 30010, Taiwan; defrosticicle@gmail.com (J.A.W.); bonbon19940827@gmail.com (C.-Y.H.); wwwu@mail.nctu.edu.tw (W.-W.W.)

**Keywords:** gold, nanotwin, preferred orientation, electrodeposition, bonding

## Abstract

We reported that highly (111)-oriented nanotwinned gold can be fabricated by periodical-reverse electroplating. The as-deposited films are shown to have a strong (111) preferred orientation, increasing with the reverse current time. The ratios of I_(111)_/I_(220)_ and I_(111)_/I_(200)_ in X-ray diffraction signals indicates a strong (111) preferred orientation. Using the advantage of the fast surface diffusion of (111) plane compared to the other planes of gold, we performed direct bonding with different thicknesses. Grain growth was observed over two films’ interfaces to eliminate the bonding interface, when annealed at 250 °C for 1 h. Shear tests were performed to gain insight on the bonding quality. All the chips failed at either the silicon substrate or substrate-adhesion layer, showing possible higher strength than the tested maximum, 40.8 MPa.

## 1. Introduction

Moore’s Law states that every 24 months [1] (later revised to 18 months by Intel executive David House) the electric components on a unit of chip area will double. However, with the decrease of line width and pitch size, back-end processes will have a hard time keeping up with front-end processes. Traditional scaling methods of solder joints will cause a metallurgical interaction between solder balls, forming intermetallic compounds, voids and porous formation in Cu_3_Sn [2], and causing a brittle interface to form. This affects the overall strength, lowering the reliability of the chip. 

Thus in order to fulfill Moore’s Law, it is suggested to implement direct bonding of metals, effectively solving the problem of side-wetting seen in solder joints. This compliments the introduction of three-dimensional integrated circuits (3D-IC) [3], where chips are stacked on top of each other, effectively lowering signal travel distance and signal delay. Various chips may be of different materials with different properties, so lowering packaging temperature is required in order to prevent damage done to the electrical components. 

Direct bonding of metals is a groundbreaking technology to be applied in the development of 3D-IC [4]. Thermal compression bonding in particular is a common method used to drive direct bonding, with the aid of increased temperatures and pressure to bond separate metals [5]. Many studies have reported successful direct bonding of various high-conductivity materials such as copper [6,7], silver [8,9,10], and gold [11,12]. However, each material faces unique challenges and difficulties based on its inherent qualities. Copper, being the most widely used for its high conductivity and low cost, faces oxidation issues during processing [13]. Silver, with the highest conductivity of all metals, forms voids across the interface when bonded at elevated temperatures [8]. 

Thus this study takes gold into consideration. Interest in gold has been rising in recent years as a suitable material to aid or even replace bumps in chip packaging [14,15]. With acceptable conductivity and extremely strong resistance to oxidation, its stability makes it an ideal candidate for application in bump bonding. 

To improve the mechanical properties of gold and give its characteristics an advantage over regular or bulk gold, it is possible to fortify the microstructure. The implementation of nanotwinned microstructure is applied. Nanotwinned microstructure has proven to possess many strong traits, such as high strength [16], high conductivity [17] and high electromigration resistance [18], and thus is implemented with the fabrication of thin gold films for further study.

To reach the greatest potential in processing, we apply electroplating methods over sputtering deposition to fabricate thin gold films. Sputtered gold films have already been widely studied [19,20], but are limited to very thin films (submicron), and have poor performance in trench and via filling. For the application in interconnects, electrodeposition of Au will be a better approach because it can be deposited in the designated patterns, thus minimizing waste of Au materials. In addition, the electrodeposition technique can fill the trenches and via with high aspect rations, with a deposition rate that is higher than that of sputtering. Therefore, electroplating is preferable to sputtering for the application in interconnects. Electroplating methods have yet to be widely researched in gold thin film deposition, but have a huge potential, and are otherwise widely researched in various fields [21,22]. This method is preferable for two reasons: (1) it is much more compatible with trench/bump pattern deposition in packaging processes, and (2) we can control the waveforms to produce the specific desired microstructures. 

There are yet to be reports on electrodeposited gold films with highly (111)-preferred orientations with nanotwinned structures. The current focus is on (111)-preferred oriented surfaces, due to them having the fastest surface diffusion rate of all face-centered cubic (FCC) planes [23], which creates more advantageous characteristics in the application of direct bonding. We aim to not only fabricate gold thin films with nanotwinned structures via electrodeposition, but also to fabricate (111)-preferred oriented surfaces. We also hope to continue to perform direct bonding at a low pressure, while maintaining a low bonding temperature without lowering bonding strength [24]. 

In this study we focus on the experiment of highly (111)-oriented gold films fabrication via electroplating and its application in direct bonding. The high surface diffusion rate of the (111) plane of gold is the main driving factor for interface removal, and can facilitate the direct bonding in joints. It is shown to be possible to achieve different thicknesses, and grain growth is observed to occur after successful bonding. We performed a shear stress test to better understand the bonding strength.

## 2. Materials and Methods

Preparation of the seed layer wafer was done by first sputtering a 100-nm-thick titanium layer as an adhesion/barrier layer, followed by a 100-nm-thick (111)-preferred orientation gold layer as the seed layer on top of a silicon oxide wafer. The wafers were then cut into small pieces of 1 × 3 cm^2^. 

Before electroplating, the substrates were first cleaned with acetone and isopropyl alcohol, followed by deionized water to remove potential organic material and finally immersed in citric acid to remove possible oxides. The electroplating parameters were as follows: With a platinum–titanium mesh as the anode and the sample as the cathode, we performed electroplating with a Keithley 2601A model source meter. The electroplating bath is distributed by Tanaka, Tokyo, Japan (MICROFAB Au100), consisting of 10 g/L gold (added as Na_3_Au(SO_3_)_2_) dissolved in nitric acid (150 mL/L), hydrochloric acid (150 mL/L), and deionized water (700 mL/L). Highly (111)-oriented gold films were fabricated via the periodical-reverse method. Electroplating was carried out at room temperature with an on-time current density of 0.5 ASD (amperes per square decimeter) and an off-time reverse current of 0.125 ASD. The total on-time deposition minus the total off-time etching accumulated to deposition times of 7 and 15 min, resulting in thicknesses of 2 and 4 µm, respectively. The electrodeposited area was 1 × 2 cm^2^. A stirring rate of 1200 rpm was applied. We tested samples with an on-off time ratio of 40 ms:4 ms and 40 ms:20 ms for comparison of short and long reverse currents.

This experiment uses the method of thermal compression bonding to act as the bonding mechanism. The samples were cut into 3 × 3 mm^2^ pieces, immersed in acetone, and underwent ultra-sonication treatment for 5 minutes. The bonding method applied was based on the method in Liu et al.’s research [6], by placing the pieces face to face on a specially designed holder, fastened with a metal screw to apply pressure. The applied pressure was measured to be approximately 0.78 MPa, acquired from a force gauge. The holder was then placed into a vacuum oven (Thermo Electron Corporation, Waltham, MA, USA), with a vacuum degree of 10^−3^ torr. The oven was heated to 250 °C for 1 h, then cooled.

A scanning acoustic tomography (SAT) and die shear test experiment of the bonded samples was conducted to observe the effective bonded area. A 3 × 3 mm^2^ die was bonded on top of a 1 × 1 cm^2^ chip at the confirmed grain growth parameter (250 °C for 1 h). A total of 6 samples then underwent a simple shear test with a shear load of max. 100 kg applied at 100 µm/s.

The as-deposited gold films were then prepared into 1 × 1 mm^2^ samples and analyzed with X-ray diffraction (XRD, Bruker D2 with Cu target, Billerica, MA, USA) and electron back-scattering diffraction (EBSD, JEOL JSM-7800F, Tokyo, Japan) to acquire its surface preferred orientation and average grain size. The films and bonding interface were then observed with a focused ion beam (FIB, Tescan Gaia3, Brno, Czech Republic), and twin spacing was observed by a scanning transmission electron microscope, (STEM, JEOL JEM-F200, Tokyo, Japan). Surface roughness was tested with an atomic force microscope (AFM, Innova AFM, Billerica, MA, USA). SAT (Hitachi FS300II, Tokyo, Japan) was tested at Materials Analysis Technology Inc., Hsinchu, Taiwan. Shear testing was performed with a bond tester (Dage 4000 bondtester, Aylesbury, UK). Surface analysis of the surfaces of the post-shear tests was done by an energy dispersive analysis X-ray system (EDAX, OIM, Mahwah, NJ, USA).

## 3. Results and Discussions

**Figure 1:**
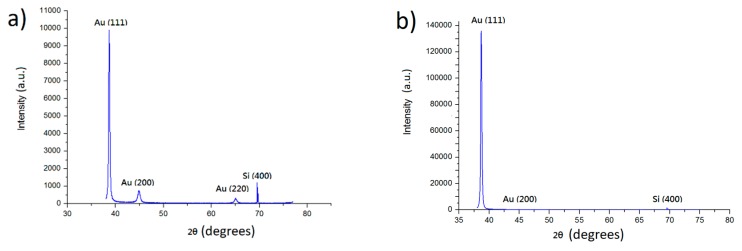
X-ray diffraction patterns for the electroplated Au films of (**a**) on-off time 40 ms:4 ms; (**b**) on-off time 40 ms:20 ms.

XRD figures show the gold films have a strong (111)-preferred orientation. Figure 1a shows the results of an on-off time of 40 ms:4 ms, while Figure 1b shows results for an on-off time of 40 ms:20 ms. The reason why results of longer reverse currents show a higher (111) signal is primarily due to reverse current being an act of etching the surface. The (111) surface has a much lower surface energy compared to other surfaces, thus remaining intact while the other surfaces are etched away after longer reverse currents. Calculations of the intensity ratios are shown in Table 1. 

**Figure 2:**
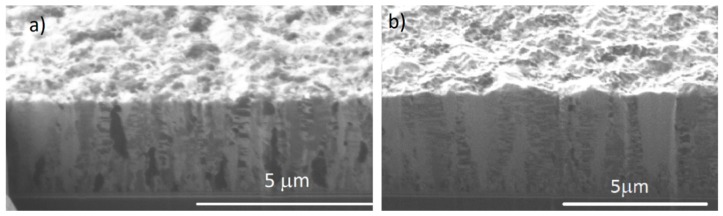
Cross-sectional focused ion beam (FIB) images showing the microstructures for the Au film deposited of (**a**) on-off time 40 ms:4 ms (**b**) on-off time 40 ms:20 ms. Thicknesses are approx. (**a**) 3.4 and (**b**) 4.8 µm.

Cross-sectional FIB images of the electrodeposited films show the microstructure of the gold films for the 40:4 and 40:20 samples, respectively. A nanotwinned structure can be observed, having a columnar formation of incoherent twins stemming from the bottom of the sample. A 0.5–1 µm thick transition layer can be also observed at the bottom, as shown in Figure 2a,b.

**Figure 3:**
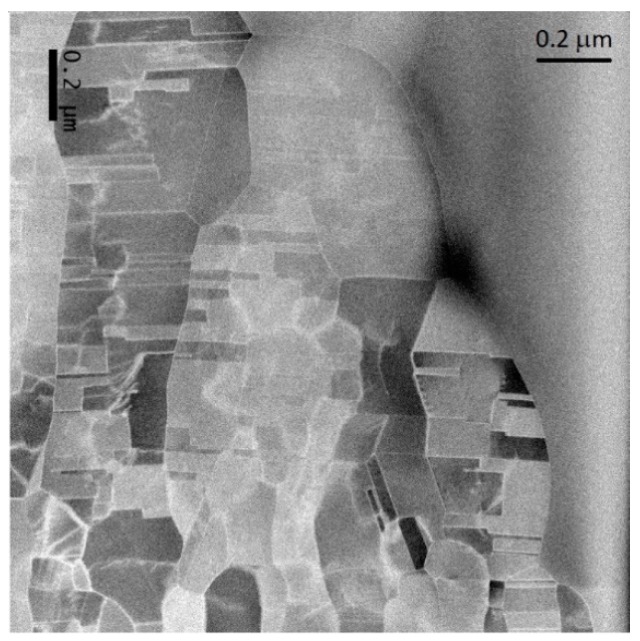
Scanning transmission electron microscope (STEM) image of twinned structure. Twins spacing is measured to be approx. 30–40 nm wide.

Scanning transmission electron microscopy (STEM) analysis on the microstructure of the films was performed to observe the twinned structure under a better resolution, as shown in Figure 3. Twins spacing ranged from 30–40 nm, showing large and small twins. The twin formation is parallel to the film with many twins of incoherent structures.

**Figure 4:**
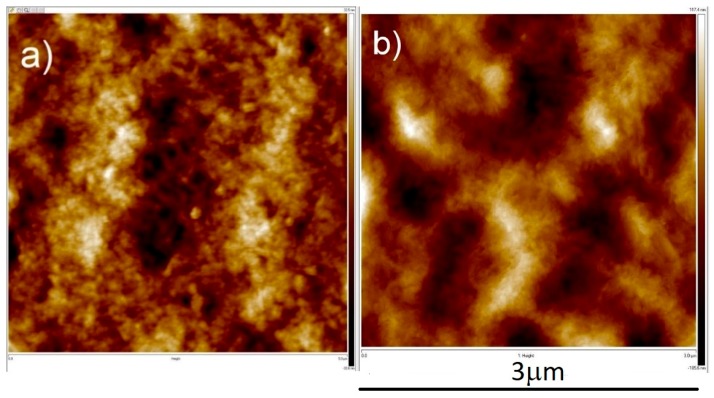
Atomic force microscope (AFM) results of area of 3 × 3 µm^2^, RMS roughness are (**a**) 9.6 nm for 40 ms:4 ms, and (**b**) 32.8 nm for 40 ms:20 ms.

AFM observations with a scanning area of 3 × 3 µm^2^ show a surface roughness (in root mean square) of the samples to be (a) 9.6 and (b) 32.8 nm, for 40:4 and 40:20 samples, respectively, as seen in Figure 4a,b. A longer reverse cycle length will cause a rougher surface, as reverse-current is basically an etching process which removes planes other than (111) for a longer period of time. We decided to use the parameters of on-off time of 40 ms:20 ms for further testing, for its high (111)-preferred orientation, compared to that of on-off time of 40 ms:4 ms, as seen in Table 1. Higher (111)-preferred oriented surfaces will be of greater aid in direct bonding, spurring surface diffusion.

**Figure 5:**
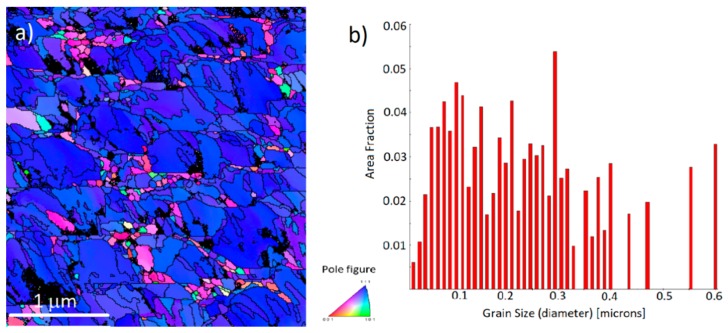
(**a**) Plan-view electron back-scattering diffraction (EBSD) showing surface orientation of gold films, with approx. 80% of the surface showing (111)-orientation. (**b**) Grain size distribution, with average grain size of 231 nm.

Plan-view EBSD data of the gold films shows a large ratio of (111)-orientation, confirmed by the intensity distribution pole, indicating that a high percentage of the surface area is (111)-oriented. Further analysis by software shows that approximately 80% of the as-deposited surface is (111)-oriented, matching with the XRD results, and can be seen in Figure 5a. Further analysis of the EBSD data shows the surface grain size distribution, having the average grain size to be around 200 nm, as shown in Figure 5b. The largest grain observed was over 600 nm, while the smallest was approximately 10 nm (which can safely be defined as a sub-micron crystal).

**Figure 6:**
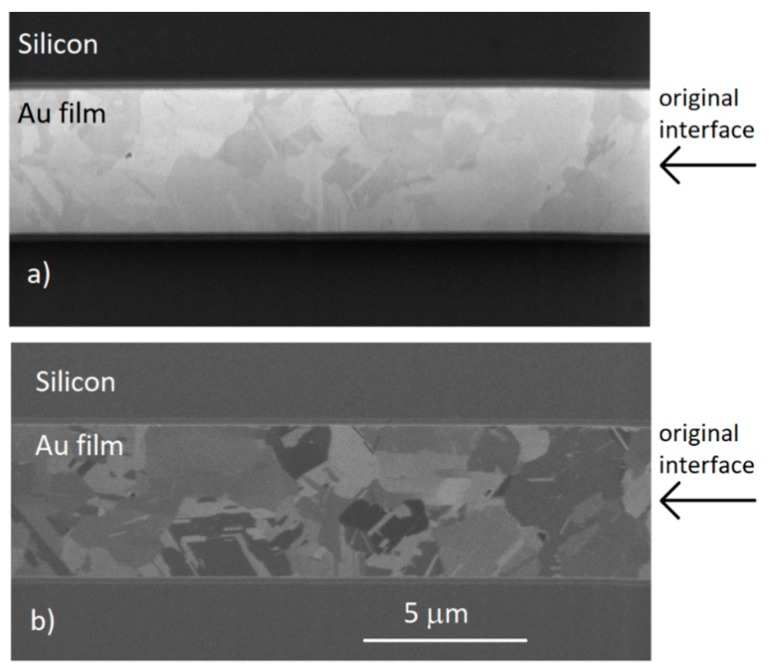
Cross-sectional (**a**) electron beam and (**b**) ion beam images of bonded 4 µm samples.

**Figure 7:**
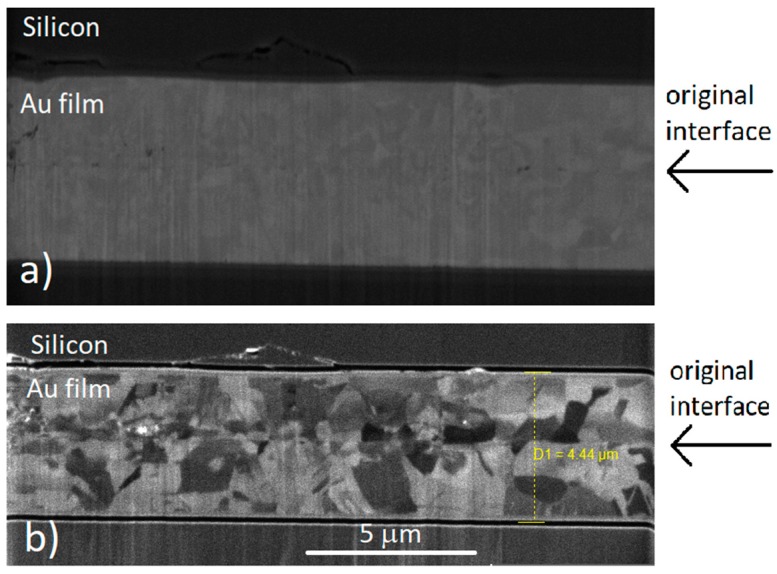
Cross-sectional (**a**) electron beam and (**b**) ion beam images of bonded 2 µm samples.

To compare results with previous studies on DC electroplated films performed in our lab, bonding tests were performed with 4 µm and 2 µm thick films. The films were electroplated with the 40:20 cycle ratio. Films 4 and 2 µm thick bonded at 250 °C for 1 h, both showing grain growth across the interface, as seen in Figure 6a,b and Figure 7a,b. The figures show the electron beam and ion beam image results. The columnar structure has disappeared, undergoing grain growth. Grains grew up to the size of 3–4 µm, increasing from the original size of approx. 0.2 µm. This shows that thinner films are able to perform as well as thicker films, successfully eliminating the original interface. 

**Figure 8:**
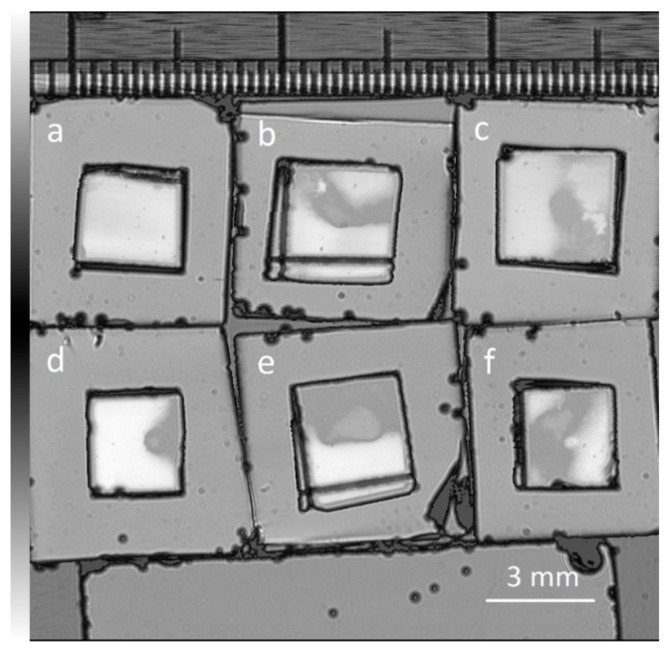
Scanning acoustic tomography (SAT) results of bonded die-chip samples, with greyed area showing bonded areas. Sample (a) was ignored in future tests due to its insufficient bonding area.

In order to accurately measure the shear force, it is essential to first confirm the actual bonded area for calculations. Considering that the thin gold films are produced in a lab, and are not fully flat surfaces, the overall bonding quality needs to be confirmed. SAT tests were done to calculate the bonded area, as shown in Figure 8. We bonded several samples with the 40:20 cycle ratio and 4 µm thick parameters for shear tests. The shear test results are shown in Table 2. For sample (a) the bonded area was too small and ineffective, showing a low strength value after the shear test. Therefore, we ignored this sample in further analysis. The observed force was then divided by the effective area to obtain the strength of the bonded samples. 

**Figure 9:**
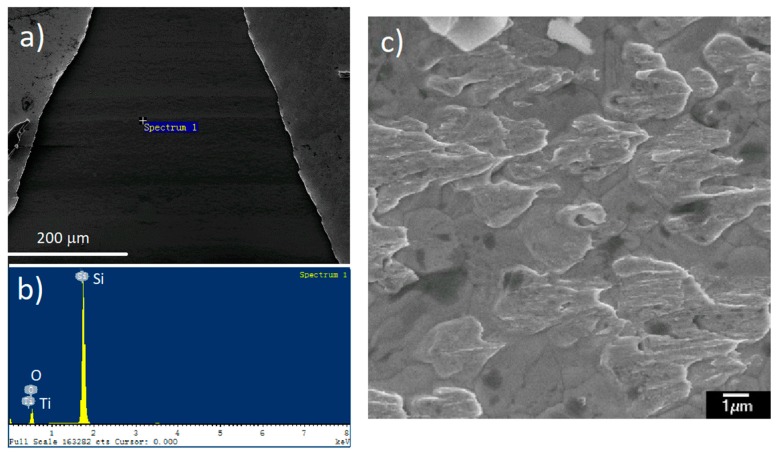
(**a**,**b**) EDAX results of separated surface, showing exposed silicon oxide below after shear test; (**c**) Plan-view SEM image of stretched grains on fractured surface.

The observed force values are the maximum detected value during the test. The bond-testing instrument will completely halt all testing once the die leaves the chip, or if there is any detection of force drop. The results of all six samples tested in this experiment are measured in two ways: (1) when film-substrate undergoes separation on the chip side, with the chip adhesion layer separating from its substrate, as proven in EDAX results Figure 9a,b, or (2) when the silicon substrate cracks. The shear force values oscillate due to the films not being fully flat surfaces, possibly lowering bonding strength. It can be thus speculated that the true bonding strength of the experiment should be higher than the maximum tested shear strength, 40.8 MPa, at a minimum. Observation of the samples that cracked at the silicon chip show partial areas with extended grains, as shown in Figure 9c. It can be speculated that the shear force causes the film surface to undergo a “stretching” mechanism.

The nanotwinned structure formation is largely determined by the on-time and off-time in the pulse electrodeposition waves. Pulse-on time generates a tensile strength, pulse-off time allows a relaxation. Twin boundaries are formed for their preferred state to release strain during film growth once strain starts to exceed a certain value [25,26]. In FCC structured systems, stress-relaxed nanotwinned structures can be of lower energy than strained FCC structures, and are thus preferred. The twinned formation would greatly contribute to the stress relaxation. 

For the surface diffusivity calculations during the bonding process the Arrhenius’ equation D = D_0_exp(−E_a_/k_B_T) is taken into calculation, where D is the diffusivity, D_0_ is the diffusion constant depending on different materials and travel path, E_a_ is the activation energy, k_B_ is the Boltzmann constant, and T is the absolute temperature. Of the calculated results D_0_, E_a_ are taken from study of P.M. Agrawal et al. [23]. Through this equation we can calculate the diffusivity of gold atoms on different surfaces, and obtain the different diffusion speed of preferred orientations. As calculated in Table 3, with the temperature at 523 K, D_(100)_ is 1.02 × 10^−9^ cm^2^/s, D_(110)_ is 3.25 × 10^−12^ cm^2^/s, and D_(111)_ is 5.93 × 10^−6^ cm^2^/s. With the diffusion rate of (111) surface being much higher than that of the other surfaces, this encourages surface diffusion, forming large grain growth in the process. 

**Figure 10:**
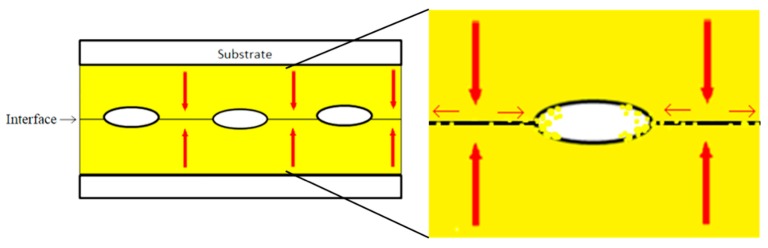
Schematic diagram of bonding mechanism at interface; a stress gradient forms between that of contacted areas (higher gradient) and voids (lower gradient), with the atoms then driven to diffuse along the interface to “fill” voids. The high surface diffusivity of the (111) surface is the main contributor to this phenomenon.

The surface diffusion path follows a creep model proposed by Liu et al. [6]. Distribution of cavities along the contacted interface is assumed. The contacted area surrounding the cavity is under a compressive stress, while the free surface, or non-contacted area, of the cavity has no normal stress. The stress will form a gradual drop, in turn becoming a stress gradient. Due to a creep effect, when a compression stress is applied, the stress gradient between contacted grains and cavity voids will become a driving force, as shown in schematic Figure 10. This drives atoms to diffuse along the surface, or in this case, the (111) surface, “closing” the cavity to perform a successful direct bonding.

It can therefore be speculated that the bonding process is split into two parts. When compression is applied, the films will first undergo a plastic deformation from the applied force. The force will first cause the surfaces to deform and fill in larger areas of the cavities on the interface caused by surface roughness. After the temperature rises to the higher bonding temperature, the film will undergo the creep transformation. With the stress gradient present, gold atoms would then fill the voids amongst the interface due to the nature of its fast surface diffusion on the (111) surface.

## 4. Conclusions

Electroplating using periodical-reverse method with an on-time current density of 0.5 ASD and off-time current density of 0.125 ASD allows for the fabrication of highly (111)-oriented nanotwinned gold films. XRD and EBSD results confirm that the surface has a strong (111)-preferred orientation. The surface roughness of the gold films is shown to be lower in films with a shorter reverse current time, with the surface roughness of on-to-off time of 40 ms:20 ms to be approximately 32.8 nm. Bonding results show successful grain growth, and no oxides forming at the surface. Cross-sectional figures of the thin film show a columnar twinned structure, with grain size to be approximately 0.2 µm^2^. Films can be bonded at a vacuum of 10^–3^ torr under a pressure of approximately 0.78 MPa and a temperature of 250 °C for 1 h.

Bonded results with different thicknesses show a grain growth when annealed at 250 °C for 1 h. Grains can grow up to 3–4 µm^2^, from their original size of 0.2 µm^2^. Grains also grow across the interface, filling in any voids. From the shear test, bonding strength is speculated to exceed 40.8 MPa, as a conservative estimate. Currently all samples are shown to fail at either the film–substrate layer of the bonded chip, or at the cracking of the silicon substrate. The strength is thus concluded to be at least larger than the tested value. The diffusion rates of different surfaces show that the (111)-preferred oriented surfaces can greatly enhance diffusion speed, swiftly filling up voids on the interface, and successfully aid in the bonding process.

## Figures and Tables

**Table 1:** The calculated intensity ratios of I_(111)_/I_(200)_ and I_(111)_/I_(220)_ of X-ray diffractions for 40 ms:4 ms and 40 ms:20 ms gold films.

On-Time:Off-Time	I_(111)_/I_(200)_ Ratio	I_(111)_/I_(220)_ Ratio
40 ms:4 ms	13.5	39.6
40 ms:20 ms	107.5	168.5

**Table 2:** Shear testing done on die-chip samples. Force detected was divided by calculated area to obtain the shear strength. Sample (a) bonded area was too insignificant to be calculated.

Sample	Bonded Area (mm^2^)	Force (N)	Strength (MPa)
a	--	18.96	--
b	4.258	99.38	23.24
c	5.874	239.57	40.79
d	6.191	191.69	30.96
e	6.784	124.92	18.41
f	7.616	217.63	28.58
Average	6.14	174.63	60.19
Std. dev	1.25	28.41	8.45

**Table 3:** Diffusion rates of different surfaces for gold at 250 °C [7].

Parameters	E_a_ (eV)	D_0_ (cm^2^/s)	D (523K) (cm^2^/s)
(100) surface	0.64	0.0015	1.02 × 10^−9^
(111) surface	0.12	0.000085	5.93 × 10^−6^
(110) surface	0.86	0.0063	3.25 × 10^−11^
